# Supplementary material for: NKG2C+ NK Cells for Immunotherapy of Glioblastoma Multiforme
Source: Int J Mol Sci. 2022 May 23;23(10):5857. doi: 10.3390/ijms23105857 (PMC9148069; doi:10.3390/ijms23105857)
Supplement: Supplementary file 1 [file ijms-23-05857-s001.zip › ijms-1725600-supplementary.pdf]

International Journal of
Molecular Sciences

MDPI

Article

NKG2C+ NK Cells for Immunotherapy of Glioblastoma Multiforme

Shafiq Murad [1], Susanne Michen [1], Alexander Becker [1], Monika Füssel [2], Gabriele Schackert [1,3], Torsten Tonn [5], Frank Momburg [6,7] and Achim Temme[1,3,4,*]

1 Department of Neurosurgery, Section Experimental Neurosurgery/Tumor Immunology, University Hospital Carl Gustav Carus, TU Dresden, Fetscherstr. 74, 01307 Dresden, Germany; shafiq.murad@ukdd.de (S.M.); susanne.michen@ukdd.de (S.M.); alexander.becker@ukdd.de (A.B.); gabriele.schackert@ukdd.de (G.S.)
2 DKMS Life Science Lab GmbH, St. Petersburger Str. 2, 01069 Dresden, Germany; fuessel@dkms-lab.de
3 German Cancer Consortium (DKTK), Partner Site Dresden and German Cancer Research Center (DKFZ), 69120 Heidelberg, Germany
4 National Center for Tumor Diseases (NCT/UCC), 01307 Dresden, Germany
5 Department of Transfusion Medicine, Medical Faculty Carl Gustav Carus, Technische Universität Dresden, Fetscherstr. 74, 01307 Dresden, Germany; t.tonn@blutspende.de
6 Antigen Presentation and T/NK Cell Activation Group (D121), German Cancer Research Center (DKFZ), Im Neuenheimer Feld 280, 69120 Heidelberg, Germany; f.momburg@dkfz.de
7 Clinical Cooperation Unit "Applied Tumor Immunity" (D120), German Cancer Research Center (DKFZ), Im Neuenheimer Feld 280, 69120 Heidelberg, Germany
* Correspondence: achim.temme@uniklinikum-dresden.de; Tel.: +49-351-458-7011

Supplementary Data

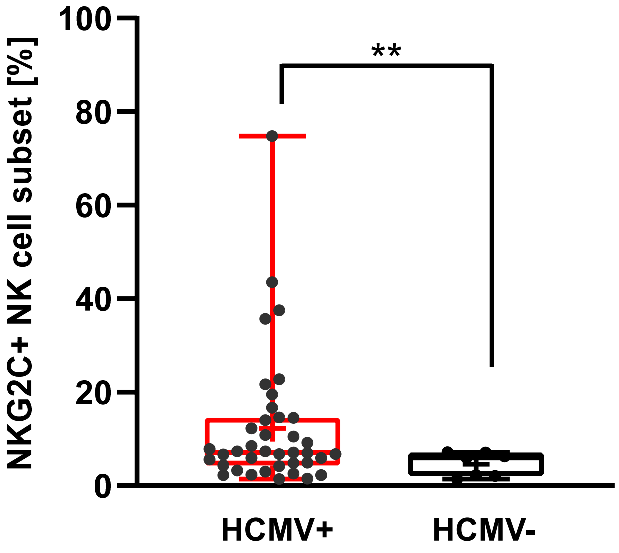

Figure S1: Assessment of NKG2C+ NK cells subsets from peripheral blood. Purified CD56+/CD3- NK cells were stained for NKG2C. HCMV status was determined by serological analysis. Mean expression levels of the NKG2C+ NK cell subset was compared between HCMV-seropositive (HCMV+) (n=39) and HCMV-seronegative (HCMV-) (n=8) donors. Dots and the horizontal lines inside the Whisker boxes indicate the mean and the median, respectively. **p<0.01.

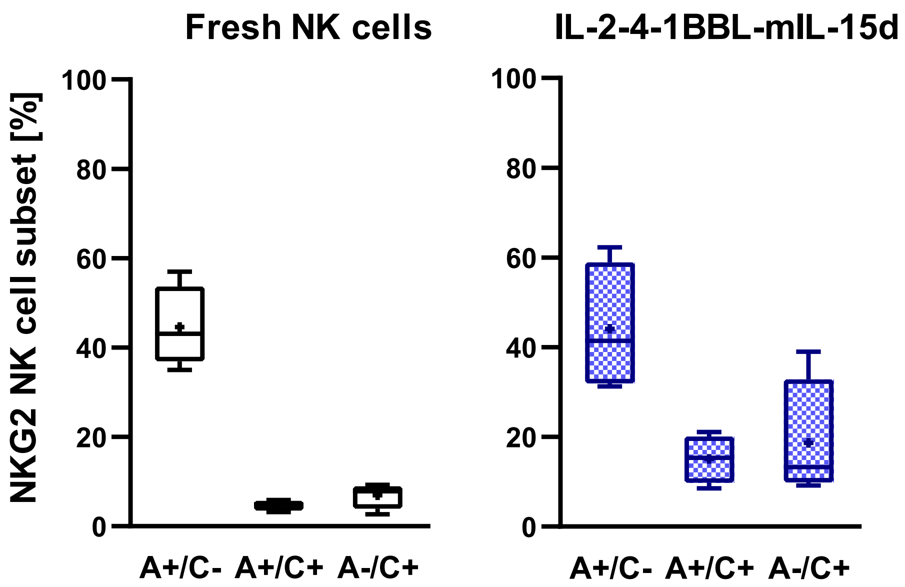

Figure S2. Flow cytometry analysis of NKG2 NK cell subsets in fresh NK cells and NK cells after 14 days expansion using PC3^{PSCA}-IL-2-4-1BBL-mIL-15d feeder cells. Left: Fresh, purified CD56+/CD3- NK cells were analyzed for their NKG2A and NKG2C expression (n=4). Right: Analysis of NKGA single-positive, NKG2A/NKG2C double-positive and NKG2C single-positive subsets in corresponding expanded NK cells. Dots and the horizontal lines inside the Whisker boxes indicate the mean and the median, respectively.

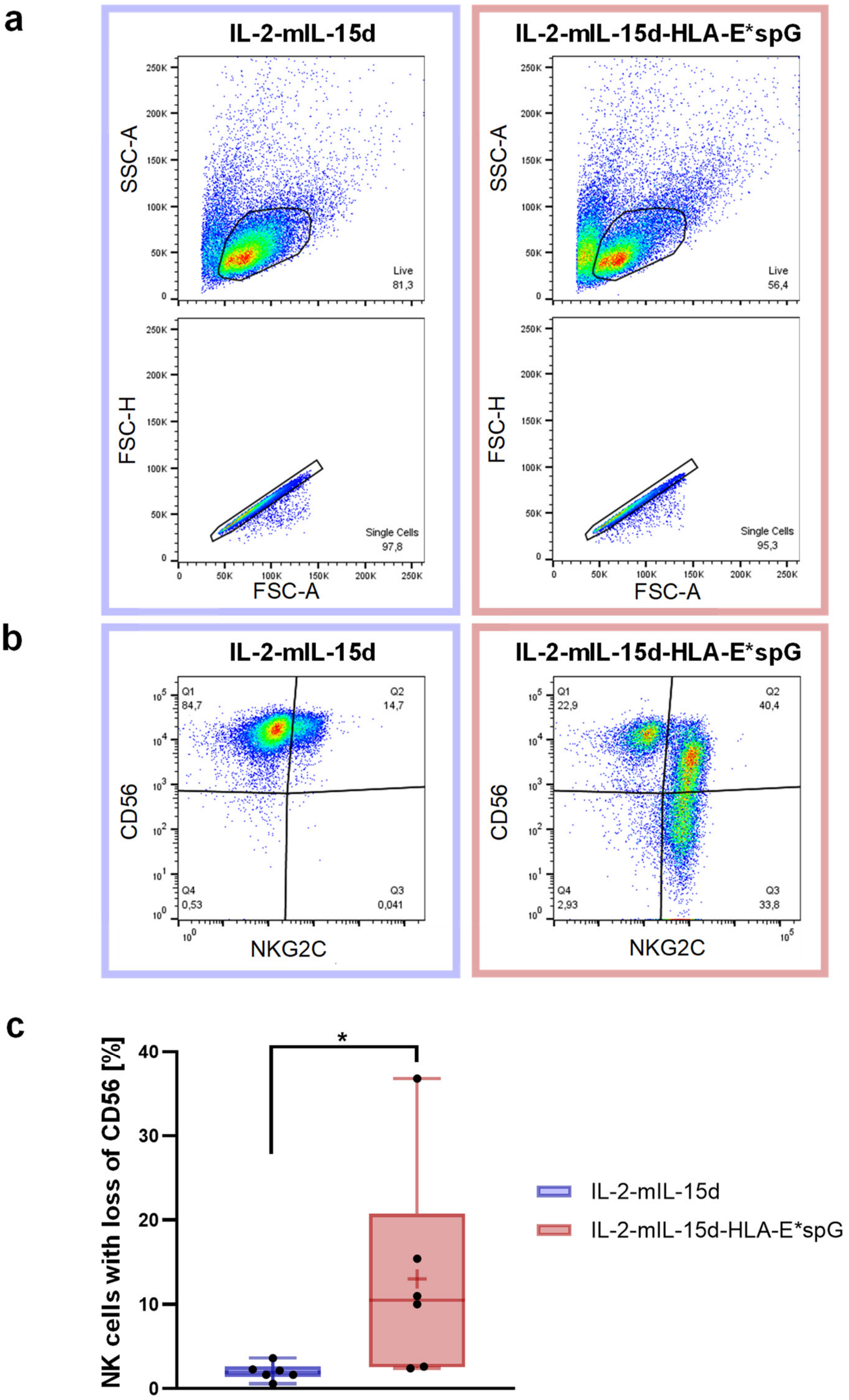

Figure S3. Shift to CD56[dim] and appearance of NK cells with further decreased MFI for CD56 after expansion with PC3[PSCA]-IL-2-mIL-15d-HLA-E*spG feeder cells. (**a**) Depicted is the gating strategy for expanded living NK cells and singlets of one representative donor analyzed by flow cytometry. Note increased cell debris and dead cells when NK cells are expanded by PC3[PSCA]-IL-2-mIL-15d-HLA-E*spG feeder cells. (**b**) Depicted are dot blots of gated NK cells of the above-mentioned donor simultaneously stained for CD56 and NKG2C. Note that NK cells expanded with PC3[PSCA]-IL-2-mIL-15d are mostly CD56[bright]. NKG2C+ NK cells expanded with PC3[PSCA]-IL-2-mIL-15d-HLA-E*spG feeder cells show a shift towards the CD56d[im] phenotype (Q2) and additionally contain a fraction of NKG2C+ NK cells with further decreased MFI for CD56 (Q3). (**c**) Analysis of the NK cell fraction with decreased MFI for CD56 expanded with PC3[PSCA]-IL-2-mIL-15d and PC3[PSCA]-IL-2-mIL-15d-HLA-E*spG feeder cells, respectively. Dots and the horizontal lines inside the Whisker boxes indicate the mean and the median. n=6. $p<0.05$.

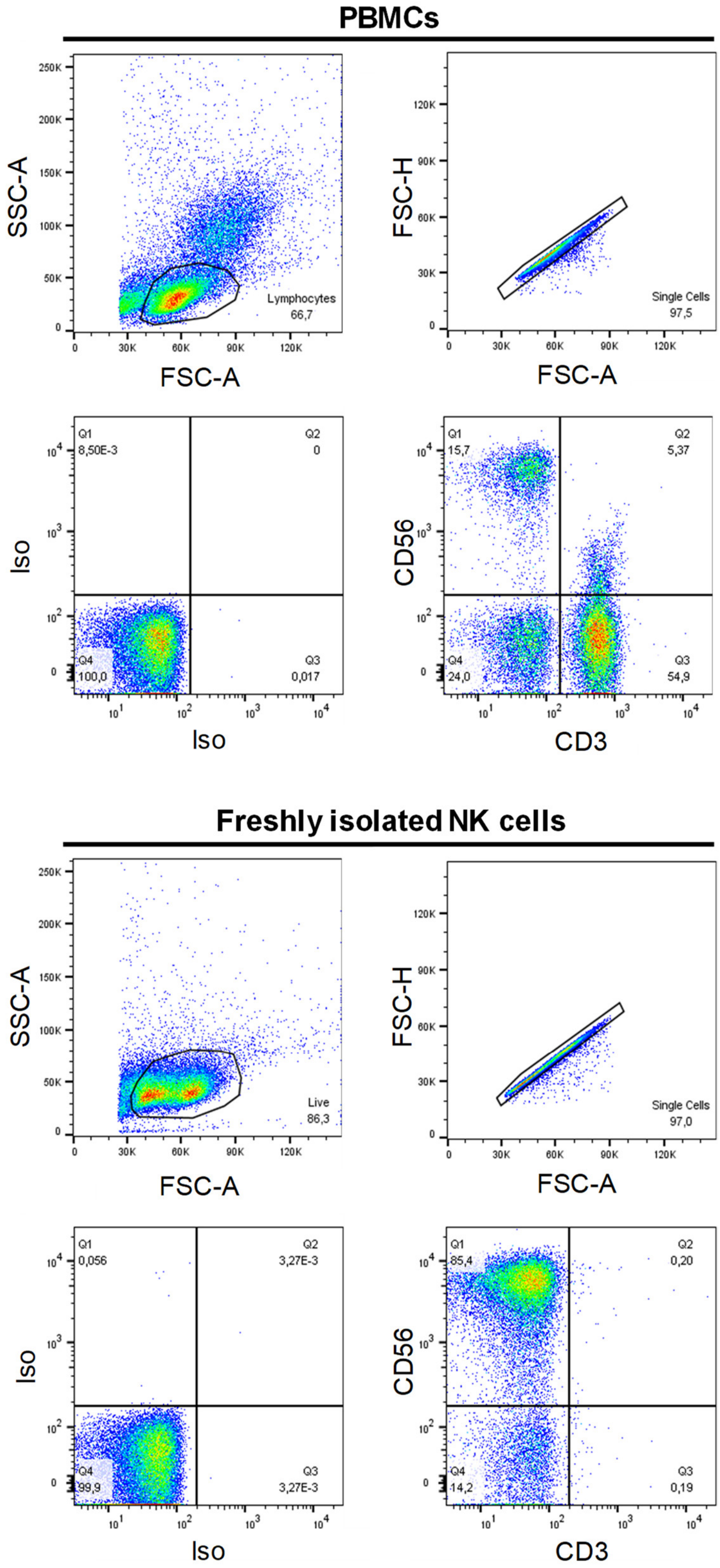

Figure S4. Isolation of NK cells using MACS-assisted negative depletion. Depicted are the flow cytometry gating strategy and analysis of one representative donor using PBMCs and after NK cell isolation. Dot blots showing gating strategy for the exclusion of dead cells and cell doublets. Isotype controls are included. The frequency of CD56+/CD3- NK cell, CD56+/CD3+ NKT cell and CD3+/CD56- T cell subsets is depicted in the respective quadrants.
